# The cost of health professionals' brain drain in Kenya

**DOI:** 10.1186/1472-6963-6-89

**Published:** 2006-07-17

**Authors:** Joses Muthuri Kirigia, Akpa Raphael Gbary, Lenity Kainyu Muthuri, Jennifer Nyoni, Anthony Seddoh

**Affiliations:** 1 World Health Organization, Regional Office for Africa, Brazzaville, Congo; 2 School of Public Health, Kenyatta University, Nairobi, Kenya; 3 Health Financing and Social Protection Unit, Division of Health Systems and Services Development, World Health Organization Regional Office for Africa, D'Joue Road, P.O. Box 06, Brazzaville, Congo

## Abstract

**Background:**

Past attempts to estimate the cost of migration were limited to education costs only and did not include the lost returns from investment. The objectives of this study were: (i) to estimate the financial cost of emigration of Kenyan doctors to the United Kingdom (UK) and the United States of America (USA); (ii) to estimate the financial cost of emigration of nurses to seven OECD countries (Canada, Denmark, Finland, Ireland, Portugal, UK, USA); and (iii) to describe other losses from brain drain.

**Methods:**

The costs of primary, secondary, medical and nursing schools were estimated in 2005. The cost information used in this study was obtained from one non-profit primary and secondary school and one public university in Kenya. The cost estimates represent unsubsidized cost. The loss incurred by Kenya through emigration was obtained by compounding the cost of educating a medical doctor and a nurse over the period between the average age of emigration (30 years) and the age of retirement (62 years) in recipient countries.

**Results:**

The total cost of educating a single medical doctor from primary school to university is US$ 65,997; and for every doctor who emigrates, a country loses about US$ 517,931 worth of returns from investment. The total cost of educating one nurse from primary school to college of health sciences is US$ 43,180; and for every nurse that emigrates, a country loses about US$ 338,868 worth of returns from investment.

**Conclusion:**

Developed countries continue to deprive Kenya of millions of dollars worth of investments embodied in her human resources for health. If the current trend of poaching of scarce human resources for health (and other professionals) from Kenya is not curtailed, the chances of achieving the Millennium Development Goals would remain bleak. Such continued plunder of investments embodied in human resources contributes to further underdevelopment of Kenya and to keeping a majority of her people in the vicious circle of ill-health and poverty. Therefore, both developed and developing countries need to urgently develop and implement strategies for addressing the health human resource crisis.

## Background

Most countries in the African Region of the World Health Organization (WHO) continue to experience the loss of a sizeable number of highly skilled health professionals (physicians, nurses, dentists, and pharmacists) by their migration to developed countries. There are three categories of emigrants: scientific trainees (Master's and PhD level) who go overseas for training but fail to return upon completion of their studies; health professionals who obtain advanced training in developed countries, return upon completion of their studies and then emigrate after working for some duration; and health professionals who train in local institutions but emigrate upon completion of their studies and/or after working for some period of time.

Emigration results from a combination of push factors (in source countries) and pull factors (in recipient countries). The reasons for scientific researchers failing to return to their home countries after training abroad include: lack of research funding; poor research facilities; limited career structures; poor intellectual stimulation; threats of violence; lack of good education for children in home country [1,2]; and lack of the evidence-based decision-making culture, leading to lack of recognition of potential contribution of researchers to national health development.

The key push factors driving out health workers include: weak health systems; insecurity including violence at the workplace; poor living conditions; low remunerations; lack of professional development opportunities (e.g. continuing education or training); lack of clear career development paths [3]; and risk of HIV infection due to lack of appropriate protective gear when handling specimens, blood and blood products; nepotism in recruitment and promotion; political unrest/civil wars; widespread poverty; poor governance; and case overload.

Some of the factors that pull professionals to developed countries may include: availability of information, easy access to communication and technology, making it easy to find jobs or complete visa applications and process; aggressive targeted recruitment to fill vacancies in richer countries; availability of employment opportunities; better remunerations and working conditions [4]; secure and conducive living conditions; and opportunities for intellectual growth (e.g. refresher courses, access to Internet and modern library facilities).

The push and pull factors in tandem have led to brain drain of health professionals from African countries. This has exacerbated the already weak national and district health systems, making it extremely difficult for countries in the Region to achieve the United Nations Millennium Development Goals (MDGs) [5,6].

The objectives of this study were: (i) to estimate the financial cost of emigration of Kenyan doctors to the United Kingdom (UK) and the United States of America (USA); (ii) to estimate the financial cost of emigration of nurses to seven Organization for Economic Co-operation and Development (OECD) countries (Canada, Denmark, Finland, Ireland, Portugal, UK, USA); and (iii) to describe other losses from brain drain.

## Methods

The data on the cost of non-boarding primary and secondary education were obtained from one non-profit religious mission school in Nairobi, Kenya. The public primary and secondary schools are heavily subsidized. For example, in 2003, Kenya decided to implement a free primary education policy in the entire country. Thus, use of fees charged in public schools would grossly underestimate the value of investment made by governments and society in general. The religious mission schools levy fees just enough to cover fixed and variable costs, earning neither a profit nor making a loss. At the other extreme are the private-for-profit schools that aim at making super-normal profits. The latter schools distort the resource allocation process because they reflect the overpricing of education production process. Therefore, among the three categories of schools, the fees charged by religious schools in Kenya were thought to be a closer reflection of the cost of primary and secondary education.

The primary school period is for eight years; and the secondary school is for four years. Their cost consists of tuition, lunch, transport, textbooks, stationery and uniforms. The tuition, lunch and transport fees levied by the mission schools aimed at covering the cost, not making a profit.

The data used to estimate the cost of training nurses and doctors were obtained from the University of Nairobi (the oldest national university in Kenya) medical school and its college of health sciences self-sponsored (unsubsidised) programmes. The other public university in Kenya that trains medical doctors and nurses is the Moi University. The two universities charge equal fees for training of doctors and nurses. The fees for government-sponsored students are heavily subsidized, whereas the self-sponsored students pay fees that are equal to the cost of education. Although the private universities do not train medical doctors, some of them (e.g. Methodist University and University of Eastern Africa) do train nurses. We used the fees for self-sponsored medical and nursing students in public universities as a proxy for the unsubsidised cost of tertiary education.

The nursing programme is made up of four years of training and one year of internship. The medical doctor programme consists of five years of training and one year of internship. The cost estimates were made up of unsubsidised tuition fees, accommodation and living expenses. The statistics on the number of Kenyan nurses working in OECD countries were obtained from the World Health Report 2006 [7]. The number of Kenyan doctors emigrating to various developed countries were obtained from Stilwell *et al*. [3].

To obtain the average total cost of producing a doctor (nurse) we summed up the average cost of medical school (and nursing school) and the average costs of primary and secondary schools. That gave us an approximation of the total cost of training a medical doctor and a nurse.

To obtain the returns from investment foregone by society when a doctor or a nurse emigrates, we multiplied the average total cost of educating a health professional by a compounding factor [8]. In algebraic terms, the lost return from education investment into an i^th ^doctor or nurse (*ILOSS*_*i *= *Doctor*, *nurse*_) who decides to emigrate would be:

*ILOSS*_*i *= *Doctor*, *nurse *_= *ATC*_*i*,.. _× (1 + *r*)^*t*^

Where: *ATC*_*i*,.. _= average total cost of educating a i^th ^health professional, e.g. doctor, nurse; (1 + *r*)^*t *^is the compounding factor; '*r*' is the interest rate; and '*t*' is the difference between the average retirement age and the average age at emigration. The above formula gives the accumulated value or future value of the investment made into producing a doctor or nurse in '*t*' years.

The Commercial Bank of Africa has a fixed deposit interest rate (*r*) of 6% and a mortgage rate of 16%; the East African Building Society has a fixed deposit interest rate of 8.5% and a mortgage rate of 16%; the Standard Chartered Bank has a fixed deposit interest rate of 7.2% and a mortgage rate of 16.5%; the Stanbic Bank has a fixed deposit interest rate of 5% and a mortgage rate of 14.5%; the National Bank has a fixed deposit interest rate of 6.2% and does not have a mortgage service; and the Commercial Bank of Kenya has a fixed deposit interest rate of 7.0% and a mortgage rate of 15%. The average fixed deposit interest rate is 6.65% per year. The average mortgage interest rate is 15.64% per year. The fixed deposit interest rates and the mortgage rates were obtained from the banks mentioned above by one of the authors (LHM) through face-to-face interviews with the respective customer services managers.

The past studies have attempted to estimate the cost of brain drain by taking into account only the tertiary cost and disregarding the primary and secondary school investments. We believe that this leads to underestimation of the loss of returns from investments into human resources for health that emigrate. This study takes into account the total cost of educating a health professional to be the sum of the cost of primary, secondary and tertiary education. In order to get the total future value of this investment that is lost due to brain drain, we applied the above-mentioned compounding formula to estimate the cumulative loss of future returns.

## Results

### Economic loss due to emigration of doctors

A total of approximately 167 medical doctors from Kenya work in two developed countries. Forty-four per cent of them work in the UK and 56% in the USA [3].

Table 1 presents the primary and secondary school costs breakdown per student. Table 2 presents a breakdown of the costs per nursing and medical students at the University of Nairobi.

Table 3 summarizes the cost of training a single doctor and a single nurse and the returns from investment lost when they emigrate to work outside their home country. The cost of tertiary education of a single doctor in Kenya is approximately US$ 48,169. The total cost of secondary education per student is US$ 6,865 and that for primary education US$ 10,963. Thus, the total education cost per medical doctor is US$ 65,997 (i.e. US$ 48169+US$ 6,865+US$ 10,963). That figure does not represent the loss incurred by society as a result of emigration of a single medical doctor. The real loss is the cumulative dollar value of the investment made by the Kenyan society in producing a doctor who decides to emigrate for a period of '*t*' years.

Let us assume that the average age of emigrating doctors is 30 years [3]; the average statutory pensionable age for Europe and Americas is 62 years [7]; an emigrant doctor would work for 32 years before retirement; and the current average interest rate on fixed deposits in Kenya is 6.65%. If the amount of US$ 65,997 (i.e. cost of educating one medical doctor) were put into a commercial bank for a period of 32 years at a fixed deposit interest rate of 6.65% per annum, the investment will grow to US$ 517,931. This is obtained by applying the standard compounding formula: [(initial investment) × (1+r)^t^] = [US$ 65,997 × (1+0.0665)^32^]. Therefore, on average, for every doctor that emigrates, a country loses about US$ 517,931. The economic loss incurred by Kenya as a result of the brain drain of 167 medical doctors [3] is US$ 86,494,477, i.e. 167 doctors × US$ 517,931 per doctor.

#### Sensitivity analysis of the interest rate

The lowest interest rate for fixed deposits in Kenya is 5%. Thus, when we use an interest rate of 5% instead of average interest rate of 6.65%, the returns from investing into a single doctor equals US$ 314,472, i.e. [US$ 65,997 × (1+0.05)^32^]. The return from investment in 167 doctors equals US$ 52,516,824, i.e. 167 doctors × US$ 364,041. The use of an interest rate of 5% instead of 6.65% reduces the amount of economic loss per doctor by 39.3%.

The average mortgage interest rate in Kenya is 15.64% per annum. Let us, instead, assume that US$ 65,997 is invested for 32 years at 15.64% interest rate. At that rate, the investment for one doctor would yield US$ 6,902,125, i.e. [US$ 65,997 × (1+0.1564)^32^]. And the yield for 167 doctors would be US$ 1,152,654,875, i.e. 167 doctors × US$ 6,902,125. Therefore, the use of an interest rate of 15.64% instead of 6.65% increases the amount of economic loss per doctor 13.3-fold.

#### Sensitivity analysis of the pensionable age (years)

The pensionable age (years) for the health workforce in the Americas and Europe ranges from a minimum of 55 to 67 years [7]. Assuming the minimum pensionable age of 55 years, if the amount of US$ 65,997 (i.e. cost of educating one doctor) were invested for a period of 25 years (i.e. 55–30 years), at an interest rate of 6.65% per annum, the investment will grow to US$ 330,024, i.e. [US$ 65,997 × (1+0.0665)^25^]. On the other hand, let's assume a pensionable age of 67 years. A deposit of US$ 65,997 in a commercial bank for a period of 37 years (i.e. 67–30 years), at a fixed deposit interest rate of 6.65% per annum, grows to US$ 714,621, i.e. [US$ 65,997 × (1+0.0665)^37^]. Therefore, the use of a pensionable age of 67 increases the amount of economic loss per doctor by 38%.

### Economic loss due to emigration of nurses

According to the World Health Report 2006 [7], about 1,213 nurses and midwives trained in Kenya work in seven OECD countries, i.e. 3.3% (1213/37113) of total number of nurses and midwives working in Kenya.

The tertiary cost of training one nurse in a Kenyan school of health sciences is about US$ 25,352. Since the cost of secondary education is US$ 6,865 and that for primary education is US$ 10,963, the total cost of educating one nurse is US$ 43,180, i.e. US$ 25,352 + US$ 6,865 + US$ 10,963.

Let us assume that the average age of emigrating nurses is 30 years; the average statutory pensionable age for Europe and Americas is 62 years [7]; an emigrant nurse would work for 32 years before retirement; and the current average interest rate on fixed deposits in Kenya is 6.65%. If the amount of US$ 43,180 (i.e. cost of educating one nurse) were put into a commercial bank for a period of 32 years, at a fixed deposit interest rate of 6.65% per annum, the investment will grow to US$ 338,868, i.e. [US$ 43,180 × (1+0.0665)^32^]. Therefore, on average, for every nurse that emigrates, a country loses about US$ 338,868. Applying that figure to all the 1,213 Kenyan nurses working in the seven OECD countries results in an economic loss of US$ 411,046,884, i.e. 1213 nurses × US$ 338,868 each.

#### Sensitivity analysis of the interest rate

The lowest fixed deposit interest rate in Kenya currently is 5% per annum. If we used an interest rate of 5% instead of 6.65%, the returns from investing in one nurse would yield US$ 205,750, i.e. [US$ 43,180 × (1+0.05)^32^]. The use of an interest rate of 5% reduces the amount of economic loss per nurse by 39.3%.

The average housing mortgage interest rate in Kenya is 15.64% per year. Let us, instead, assume that the amount of US$ 43,180 is invested for 32 years at 15.64% interest rate. At that rate, the investment for one nurse would yield US$ 4,515,869, i.e. [US$ 43,180 × (1+0.1564)^32^]. Therefore, the use of an interest rate of 15.64% increases the amount of economic loss per nurse 12-fold, i.e. compared to the economic loss at the average interest rate of 6.65%.

#### Sensitivity analysis of the pensionable age (years)

The pensionable age (years) for the health workforce in the Americas and Europe ranges from a minimum of 55 to 67 years [7]. Assuming the minimum pensionable age of 55 years, if the amount of US$ 43,180 (i.e. cost of educating one nurse) were put into a commercial bank for a period of 25 years (i.e. 55–30 years), at a fixed deposit interest rate of 6.65% per annum, the investment will grow to US$ 215,926, i.e. [US$ 43,180 × (1+0.0665)^25^]. On the other hand, let's assume a pensionable age of 67 years. A deposit of US$ 43,180 in a commercial bank for a period of 37 years (i.e. 67–30 years), at a fixed deposit interest rate of 6.65% per annum, grows to US$ 467,557, i.e. [US$ 43,180 × (1+0.0665)^37^]. Therefore, the use of a pensionable age of 67 increases the amount of economic loss per nurse by 38%.

## Discussion

### Key findings

The objectives of this study were: (i) to estimate the opportunity cost of emigration of Kenyan doctors to the United Kingdom (UK) and the United States of America (USA); (ii) to estimate the opportunity cost of emigration of nurses to seven OECD countries (Canada, Denmark, Finland, Ireland, Portugal, UK, USA); and (iii) to describe other losses from brain drain. The key findings were as follows:

▪ The total cost of educating a single medical doctor from primary school to university was US$ 65,997.

▪ On average, for every doctor that emigrated, a country lost about: (i) US$ 517,931, assuming a 6.65% interest rate; (ii) US$ 314,472, assuming an interest rate of 5%; and (iii) US$ 6,902,125, assuming an interest rate of 15.64%.

▪ The total cost of educating one nurse from primary level to college of health sciences was US$ 43,180.

▪ On average, for every nurse who emigrated, a country lost about: (i) US$ 338,868, assuming a 6.65% interest rate; (ii) US$ 205,750, assuming an interest rate of 5%; and (iii) US$ 4,515,869, assuming an interest rate of 15%.

▪ The application of an interest rate of 15.64% instead of 6.65% increases the economic losses resulting from emigration of a doctor and a nurse by 13-fold.

The United Nations Commission for Trade and Development (UNCTAD) has estimated that each migrating African professional represents a loss of US$ 184,000 [1]. Our study estimated economic loss incurred by African countries as a result of emigration of one doctor to be about US$ 517,931 and one nurse to be US$ 338,868. Thus, our estimated losses to Kenya as a result of emigration of a single medical doctor and a single nurse are two times and three times the UNCTAD estimate respectively. Our estimates are higher than those of UNCTAD for two reasons: (i) we take into account the investments made into production of doctors and nurses from primary school to tertiary training institutions; and (ii) we take into account the cumulative financial effects of the lost returns from investments as a result of the brain drain.

### Other losses from brain drain

When health professionals emigrate, Kenya loses far more than the cost incurred by society to educate them. This is because there are several other losses that are not captured in the education-costing methodology. Some of those losses are:

#### Loss of health services

Health professionals (especially doctors and nurses) contribute to health promotion, disease prevention, diagnosis, treatment and rehabilitation. The ratios of doctors and nurses to the population in Kenya are very low, and, as a result, medical practitioners and nurses are usually overloaded with work [9]. Thus, the emigration of doctors and nurses (and other health professionals) exacerbates the human resource shortage within the national and district health systems and reduces their capability to perform their functions (of stewardship, health financing, resource/input creation and health service production and provision) and achieve their goals of health improvement, responsiveness to client's legitimate expectations and fairness in financial contributions.

#### Loss of supervisors

Practising doctors and senior nurses normally play major roles in supervising staff in peripheral facilities (e.g. health centres, dispensaries and health posts) that serve the majority of populations. Thus, when such doctors and nurses emigrate, the supervisory capability is lost (or diminished), contributing to further weakening of the capacities of such health facilities to provide quality services to patients. This compels the staff left behind to assume greater responsibilities than they had been trained for, invariably leading to a decline in the quality of health services.

#### Loss of mentors for health sciences trainees

Practising doctors (and senior nurses) train and counsel (advise) new employees and students doing their internship. The emigration of either cadre has negative inter-generational effect on the process of health-related human capital creation in the country.

#### Loss in functionality of referral systems

The hierarchical national referral system consists of tertiary hospitals (apex), provincial hospitals, district hospitals, health centres, dispensaries, health posts and community services. It permits movement of patients from the base of the national health system to the apex and vice versa. Although the movement of patients should, in principle, be initiated by health professionals, in practice, patients move themselves up and down this system. Patients bypass the cheapest health units (health centres, dispensaries and health posts) mainly due to lack of doctors and diagnostic services [10]. Those two factors create adverse incentives for patients to bypass the cost-effective health units and to seek care in more expensive hospitals. Thus, emigration of doctors contributes to inefficiency and weakening of the referral system.

#### Loss of role models

Children often view doctors and nurses practising in communities as examples to be imitated and emulated. Thus, external migration not only robs such children of positive role models, it also negatively affects their dreams and aspirations and hence the number of children aspiring to become health professionals.

#### Loss of public health researchers

Many of the specialized doctors who emigrate are often among the very few active/published researchers that the country has. Emigration of such people stifles innovation and invention in persistent local public health problems, e.g. HIV/AIDS, tuberculosis and malaria.

#### Loss of custodian of human rights, especially in rural areas

A recent study on the status of national health research bioethics committees in the WHO African Region found that many countries did not have functional ethical review systems that protected the dignity, integrity and safety of citizens who participated in research [11]. Authors argued that health professionals who were posted in rural areas, by virtue of being the most educated, often bore the burden of assuring that the human rights of their actual and potential clients were respected and protected in the course of their clinical work and research carried out by others.

#### Loss of savings (investment capital)

In Kenya, health professionals are among the relatively better-paid persons, and thus they contribute to accumulation of national savings. Those savings are eventually loaned to entrepreneurs for investment. Thus, emigration may lead to loss of such savings, except where persons who emigrate remit their savings back to the country for investment.

#### Loss of entrepreneurs

The health practitioners, by virtue of their education and earnings, quite often set up health-related (e.g. private clinics, hospitals, pharmacies) and non-health-related businesses (e.g. retail and wholesale shops). Thus, emigration reduces the growth of entrepreneurship in affected countries and the prospects for economic growth.

#### Loss of employment opportunities

Doctors and nurses usually provide job opportunities for housekeepers, gardeners and security guards at their places of residence. Thus, emigration of practising health professionals usually results in loss of employment opportunities and income for those poor workers and their families.

#### Loss of tax revenue to government

Given the fact that health professionals are among the relatively well-paid persons in Kenya, they are also major contributors to the country's income-tax collection. Since the incomes of emigrants are not liable to tax administration systems of Kenya, emigration leads to a net loss in tax revenues.

#### Disruption of families

In some instances, due to immigration restrictions, the emigrating health professionals are not allowed to take along their families. Due to spatial distance and loneliness, some of those emigrants may choose to get new marriage partners in their countries of work. This may bring psychological and economic suffering to family members left behind in Kenya.

#### 'Internal' brain drain

The brain drain, broadly construed, not merely reduces the supply of vital health professionals in Kenya, even more seriously, it diverts the attention of those who remain from important local problems and goals [12]. These include provision of primary health care services (including health promotion and primary and secondary prevention of diseases) and promotion of problem-oriented training and research on important domestic public health issues. Such needs are often neglected as training and research get dominated by rich-country ideas as to what represents true professional excellence. Those highly educated and skilled Kenyan health professionals who do not physically migrate to developed countries 'migrate intellectually' in terms of the orientation of their activities.

#### Loss of an important element of the middle class

Arguably, physicians comprise an important segment in the social and economic make-up of the middle class. They are generally respected as being above corruption, they advocate for quality public schools, they provide a market for consumer goods, and they contribute to political, social and economic stability. Furthermore, they create demand for democratic institutions [13].

### Alleged redeeming features of movement of health workers abroad

Some middle-income countries like the Philippines train health workers for international export. The World Health Report 2006 [7] states that: "The government of the Philippines has encouraged temporary migration by its professionals in recent years and taken measures to turn remittances into an effective tool for national development by encouraging migrants to send remittances via official channels. In 2004, the Central Bank of the Philippines reported total remittances of US$8.5 billion, representing 10% of the country's gross domestic product (GDP)" (p.101).

Unlike the case of the Philippines, where the government strategically encourages temporary emigration of health workers for remittances and for acquisition of skills and expertise, the Kenyan government does not encourage health professionals to emigrate due to the large unmet need for their services, especially in rural areas where about 80% of the population lives. The relatively meagre remittances by the Kenyan health professionals working abroad are sent directly to family members and not through official treasury channels [13]. Those resources are not available for strengthening of medical (and nursing) schools and national health systems. Furthermore, although remittances are beneficial to the emigrants' family members, they may contribute to widening the gap between the rich and poor.

It has also been argued that if health workers return home, they bring significant skills and expertise back to their home countries. There is no evidence that Kenyan health professionals working abroad ever return home after working for a few years to share the knowledge and skills acquired abroad.

The 'fiscal space' (budgetary room) [14] in low-income countries, like Kenya, has often constrained them from employing all the available human resources for health. This has often resulted in the paradoxical scenario of unemployment of some cadres of health professionals amidst the large unmet need for their services. For example, around 4,000 unemployed nurses in Kenya do not constitute a real surplus since there is a large unmet need for their services [15].

Heller [14] indicates that countries like Kenya can potentially increase their "fiscal space", for example to employ the unemployed nurses, in four ways: (i) generation of additional revenues through increased taxes or strengthened tax administration; (ii) efficiency savings or reduction in unproductive expenditures, e.g. spending on defence, foreign travel or embassy expenses; (iii) domestic and/or external borrowing, e.g. sustained and predictable external grants; and (iv) printing money to finance additional government spending, which is an undesirable option since it might fuel inflation.

Even assuming that 'fiscal space' was not an issue, that there was a real surplus of nurses, and that Kenya decided to export them to developed countries, the government should try to negotiate for reimbursement of not just the training cost of US$43,180 per nurse but the lost returns from investment of between US$205,750 and US$4,515,869 per nurse. Those resources can be used to strengthen the tertiary institutions that produce human resources for health.

### Limitations of the study

Our study had some limitations:

a) Due to the unavailability of data, the study focused on only two categories of health professionals (i.e. doctors and nurses) even though there was anecdotal evidence of brain drain among other cadres.

b) The cost estimates presented in this study were only for undergraduate programmes, while many of the doctors and nurses emigrating may have had postgraduate qualifications. Thus, by not capturing the postgraduate investment, we would have underestimated the cost of training, and hence, the lost returns from investment in postgraduate training.

c) Due to lack of information on the total amounts of money remitted to Kenya by emigrant doctors and nurses, it was not possible to estimate the net loss of returns from investments resulting from emigrating health professionals. If the data on remittances were available, the net loss would have been equal to the total economic loss minus the total remittances.

### Suggestions for further research

The following aspects are in need of further research:

(a) Monitor the trends of the effects of loss of health services as a result of external migration of key cadres of human resources for health, such as specialist doctors and nurses, pharmacists and lecturers of medical and nursing schools.

(b) Establish a database of cost of primary, secondary and tertiary education of various categories of human resources for health, and cost of alternative strategies for stemming the tide of brain drain.

(c) Establish a programme for systematic monitoring of international migration of different cadres of human resources for health and tracking of remittances of income.

(d) Application of the contingent WTP approach in the valuation of the socioeconomic loss incurred by Kenya due to brain drain of different categories of human resources for health. It has also been applied in Africa to value the benefits of insecticide-treated bednets [16,17], community-based health insurance [18,19] and health outcomes of interventions against schistosomiasis [20].

(e) Identify the determinants of health staff motivation, including their health-related quality of life [21] and retention through regression analysis.

## Conclusion

Our study estimated the economic loss incurred by Kenya as a result of emigration of one doctor to be about US$ 517,931 and one nurse to be US$ 338,868. However, we suspect that the magnitude of the socioeconomic loss due to brain drain is likely to be even larger than our estimates. Therefore, there is need for more precision in the measurement of the magnitude of the socioeconomic loss due to brain drain, for use in advocacy and policy. We propose the use of Contingent Valuation to measure the benefits from investments into specific categories of human resources for health. Those benefits would be a more accurate indicator of the losses incurred by Kenya due to brain drain.

Developed countries continue to deprive Kenya of millions of dollars worth of invaluable investments made in the production of health workers. If the current trend of poaching of the scarce human resources for health (and other forms of human resources) from Kenya is not curtailed, the chances of achieving the Millennium Development Goals would remain dismal. Since the limited human resources for health are the head, heart and hands of the national and district health systems [22], the continued plunder of investments embodied in human resources contributes to: 1) the growing double burden of communicable and non-communicable diseases (by weakening health promotion and primary and secondary prevention); 2) further underdevelopment of Kenya; and 3) keeping a large proportion of the Kenyan population in the vicious circle of poverty and ill-health.

Economic arguments notwithstanding, ultimately the price of emigration of human resources for health from Kenya to developed nations is paid in unnecessary debility, morbidity, human suffering and premature death among Kenyan people. This unacceptable situation should be urgently reversed through joint action by both developing and developed countries.

## Competing interests

The author(s) declare that they have no competing interests.

## Authors' contributions

JMK did the analysis of the data and participated in the drafting of all sections of the manuscript. ARG, JN and AS participated in the drafting of the background, methods and discussion sections. LKM collected the data and participated in the background, methods and discussion sections.

## Pre-publication history

The pre-publication history for this paper can be accessed here:



## Figures and Tables

**Table 1:** Mission primary and secondary school costs per student

	**Primary school cost per student**				
	
***Year***	***Tuition cost per year *(*Ksh*)**	***Lunch cost per year *(*Ksh*)**	***Transport cost per year *(*Ksh*)**	***Cost of textbook*+ *stationary*+ *uniforms *(*Ksh*)**	***Sub-total costs *(*Ksh*)**
1	54 300	19 500	15 000	7 000	95 800
2	54 300	19 500	15 000	7 000	95 800
3	54 300	19 500	15 000	7 000	95 800
4	54 300	19 500	15 000	7 000	95 800
5	57 300	19 500	15 000	7 000	98 800
6	57 300	19 500	15 000	7 000	98 800
7	57 300	19 500	15 000	7 000	98 800
8	57 300	19 500	15 000	7 000	98 800

***Total cost *(*Ksh*)**	***446 400***	***156 000***	***120 000***	***56 000***	***778 400***

***Total cost *(*US$*)**	***6 287***	***2 197***	***1 690***	***789***	***10 963***

***Year***	**Mission secondary school cost per student**				

1	74 300	19 500	15 000	14 000	122 800
2	71 300	19 500	15 000	14 000	119 800
3	71 300	19 500	15 000	14 000	119 800
4	76 500	19 500	15 000	14 000	125 000

***Total cost *(*Ksh*)**	***293 400***	***7 8000***	***60 000***	***56 000***	***487 400***

***Total cost *(*US$*)**	***4 132***	***1 099***	***845***	***789***	***6 865***

Notes: US$1 = 71 Kenya Shillings (Ksh). The statistics in this table represent unsubsidized cost.

**Table 2:** Public University parallel programme cost per nursing student and cost per medical doctor

	**College of health sciences programme cost per nursing student**		
	
***Year***	***Tuition Cost Per year *(*Ksh*)**	***Accommodation*+ *Living expenses *(*Ksh*)**	***Sub-total cost *(*Ksh*)**
1	240 000	120 000	360 000
2	240 000	120 000	360 000
3	240 000	120 000	360 000
4	240 000	120 000	360 000
5	240 000	120 000	360 000

***Total cost *(*Ksh*)**	***1200 000***	***600 000***	***1800 000***

***Total cost *(*US$*)**	***16 901***	***8 451***	***25 352***

***Year***	**Public university parallel programme cost per medical doctor**		

1	450 000	120 000	570 000
2	450 000	120 000	570 000
3	450 000	120 000	570 000
4	450 000	120 000	570 000
5	450 000	120 000	570 000
6	450 000	120 000	570 000

***Total cost *(*Ksh*)**	**2 700 000**	**720 000**	**3 420 000**

***Total cost *(*US$*)**	**38 028**	**10 141**	**48 169**

Note: The statistics in this table represent unsubsidized cost.

**Table 3:** Summary of educational cost and lost returns from investment

**Institution**	**Doctor**	**Nurse**
Primary school cost (US$)	10 963	10 963
Secondary school cost (US$)	6 865	6 865
Tertiary institution cost (US$)	48 169	25 352

***Total Cost *(*US$*)**^***a***^	***65 997***	***43 180***

Total cost compounded at an average interest rate of 6.65% over 32 years (US$)^b^	517,931	338,868
Total cost compounded at an interest rate of 5% over 32 years (US$)^c^	314,472	205,750
Total cost compounded at an interest rate of 15.64% over 32 years (US$)^d^	6,902,125	4,515,869

Notes: ^a^This is the money invested in training of a doctor and a nurse.

^b^Is the return from investment lost (at 6.65% interest rate) when a single doctor or nurse emigrates.

^c^Is the return from investment lost (at 5% interest rate) when a single doctor or nurse emigrates.

^d^Is the return from investment lost (at 15.64% interest rate) when a single doctor or nurse emigrates.
